# Patterns of avian tree usage in the primeval temperate forests of Białowieża National Park

**DOI:** 10.1002/ece3.11138

**Published:** 2024-04-15

**Authors:** Oliwia Karpińska, Katarzyna Kamionka‐Kanclerska, Patryk Czortek, Marcin K. Dyderski, Dorota Czeszczewik

**Affiliations:** ^1^ Institute of Biological Sciences, Faculty of Science University of Siedlce Siedlce Poland; ^2^ Białowieża Geobotanical Station, Faculty of Biology University of Warsaw Białowieża Poland; ^3^ Institute of Dendrology Polish Academy of Sciences Kórnik Poland

**Keywords:** niche partitioning, spatial ecology, species co‐occurrence, tree usage, vertical stratification

## Abstract

Species distribution and resource utilization are a fundamental aspect of ecology. By analyzing the tree space usage by birds and determining the species composition of birds across different parts of trees, our study could shed light on the mechanisms shaping co‐occurrence patterns in bird communities. Therefore, our study aimed to determine the species composition of birds across different parts of trees. We investigated whether species richness differs between positions on a tree and how these positions influence the probability of occurrence of the 10 most frequently observed bird species. To achieve this, we observed birds within permanent plots in Białowieża National Park (BNP) and analyzed the distribution patterns of birds within six vertical and three horizontal sectors of trees. The compositional dissimilarity between tree sectors was assessed using detrended correspondence analysis. We employed generalized linear mixed‐effects models to examine differences in species richness. The majority of the BNP bird community was associated with the branches, while other birds occupied the tree crown trunks and the understory trunks. Species richness was the highest on branches in the crown part of trees, followed by lower species richness on trunks associated with crowns, and the lowest richness was observed on branches and trunks in the understory. These results indicate that branches in the middle and lower parts of the crown serve as avian diversity hotspots on trees, likely due to the abundance of various food sources. The differing patterns of tree usage by specific bird species may suggest the avoidance of interspecific competition for resources. The study results of tree usage by bird species obtained in the primeval forest provides a reference point for studies conducted in human‐altered woods.

## Graphical abstract

We investigate the vertical and horizontal stratification of bird species within trees in a pristine forest ecosystem. This work delves into the utilization of tree resources by various bird species and their interactions within the forest environment.

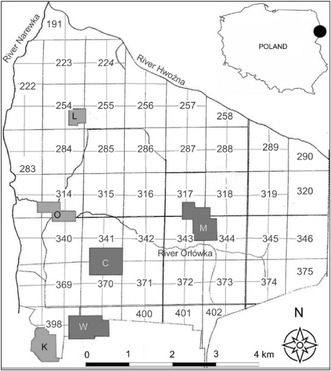

## 1 INTRODUCTION

Forests are the most productive and species‐diverse habitats amongst terrestrial areas (Mikusiński et al., 2018). Trees that create this environment are keystone structures for numerous organisms, providing them with food, shelter, and other resources (Larrieu et al., 2018). Amongst a wide range of tree‐dependent groups of organisms, birds constitute the most numerous vertebrate group (Stagoll et al., 2012). They use trees as nesting sites, roosting, and grooming places, shelters, mating arenas, etc. Each species has specific requirements such as the preferred nest site (tree hole, tree crack, forksplit, dense twigs, etc.), singing (exposed treetops, inside the crown), and drumming places (e.g., dry branches with appropriate acoustics) (Poulsen, 2002).

The width of a species' niche seems to be influenced by a complex interplay of factors including competition, resource availability, morphological traits, and foraging behaviors within their specific habitat (Saether, 1982). Birds exhibit niche partitioning within the three‐dimensional space of trees, with vertical stratification of habitats (Robinson & Holmes, 1982). Spatial avoidance of competitors is a behavior observed amongst bird species to reduce interspecific competition (Hutchinson, 1965). Such behavior, which may involve temporary changes in activity or specialization in diet, helps stabilize the bird community and promote species diversity (Chesson, 2000). Birds are capable of coexisting in the same tree space by employing various foraging techniques, utilizing different types of twigs/boughs (dead or alive), showing preferences for denser or sparser foliage, and exhibiting activity during different times (Villard & Foppen, 2018). Additionally, patterns of tree usage by birds can be influenced by habitat filters, such as the surface characteristics of tree trunks or tree diameter (Basile et al., 2020). Species such as bark feeders (e.g., *Dendrocopos* sp., *Certhia* sp.) are morphologically and physiologically adapted to forage on tree trunks compared to other species. Bark roughness can affect the availability of arthropods for insectivorous birds, while twigs of varying sizes can provide more available tree space for perching (Jackson, 1979). To illustrate the black woodpeckers *Dryocopus martius* choose trees with softer wood and higher first branch in less forested areas to reduce the energy needed for creating cavities and to lower the risk of predation (Puverel et al., 2019).

Vertical stratification in tree space is vital for stable interactions in forest bird communities. Disruptions to complex forest structures can increase niche overlap, leading to antagonistic interactions and potential species loss (Gámez & Harris, 2022). Analyzing how birds use tree structures enhances our understanding of individual species' biology, aiding conservation efforts for endangered taxa (e.g., Fricker et al., 2021). Studying bird tree use and stratification is essential for assessing ecosystem health and biodiversity (Brockerhoff et al., 2017). Changes in bird populations across tree layers indicate shifts in biodiversity and ecosystem dynamics. Identifying key habitats through bird stratification is crucial for effective conservation, preserving diverse habitats for various bird species (Basile et al., 2021). Bird tree utilization patterns also provide insights into forest vertical structure and functionality, benefiting ecologists studying forest dynamics, succession, and species interrelations (Lindenmayer et al., 2000). Despite this importance, there is a lack of comprehensive studies on the use of tree structures by breeding birds in temperate, primeval forests. Previous research has mainly focused on nesting site characteristics (e.g., Czeszczewik & Walankiewicz, 2003; Hernández, 2010; Maziarz et al., 2015) or foraging behavior (e.g., Adamík & Kornan, 2004; Alatalo, 1980; Böhm & Kalko, 2009). Observing organisms in natural habitats provides valuable insights into fundamental behavior, making research in such environments significant as a reference point for studies in human‐altered forests (Wesołowski, 2007).

The aim of our study was to determine the species composition of birds across different parts of trees, as defined by vertical and horizontal positions. Additionally, we aimed to investigate whether species richness differs between tree positions and sectors and how sector positions influence the probability of occurrence for the 10 most common bird species in BNP. In our study, we assumed that if multiple tree species occur in close proximity within a large mosaic, the identity of the tree species is less significant for birds compared to the spatial fragments (forest patches) within the horizontal and vertical gradients. Trees generally possess their own characteristics that can be expressed through sectors (i.e., particular localization on a tree) and their specific features. We did not analyze the specific effects of tree species.

## 2 MATERIALS AND METHODS

### 2.1 Study site

**FIGURE 1:**
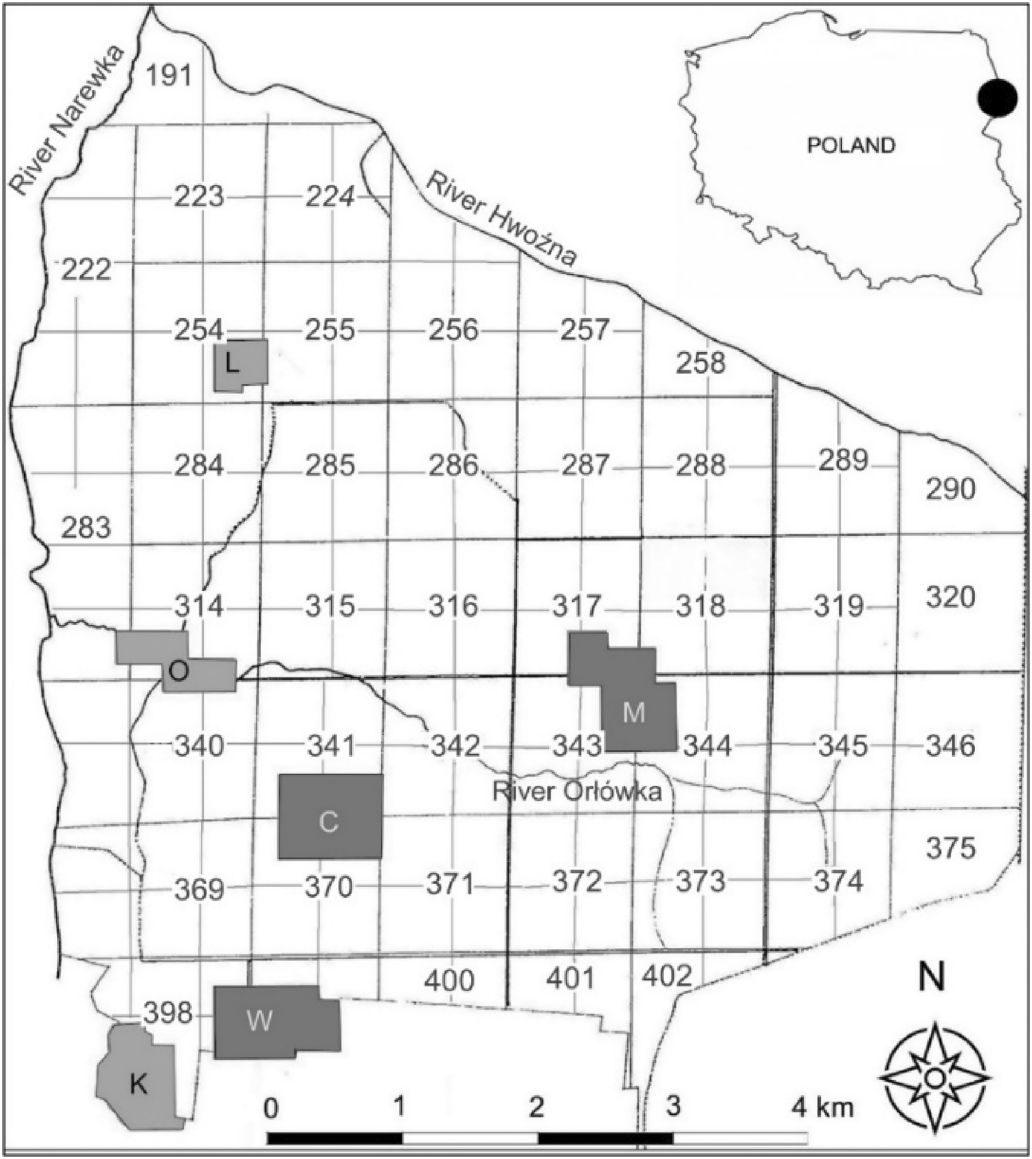
Distribution of sample plots in Białowieża National Park. Dark gray plots (W, C, M) were localized in oak‐lime‐hornbeam forest, whereas light gray plots (K, O, L) were localized in ash‐alder habitat. Forest compartments have been marked with numbers.

Our research was conducted in the Białowieża Forest, which is the last remaining primeval, lowland temperate forest in Europe. The forest is situated on the border between Poland and Belarus, between coordinates 23.52° and 24.35° E and 52.48° and 52.95° N (Figure 1). Due to minimal human impact and centuries of protection, the Białowieża Forest has maintained its natural character and extensive, uninterrupted area (Samojlik et al., 2020). The Białowieża National Park (referred to as BNP) represents the best‐preserved fragment of the forest and has been protected since 1921 (Faliński, 1986). Covering an area of 105.2 km^2^ on the Polish side, BNP is renowned for its primeval characteristics, including large tree size, multi‐story stand profiles, and a diverse array of tree species (Wesołowski & Martin, 2018). BNP encompasses three primary habitat types: mixed‐deciduous forests consisting of lime, oak, maple, and ash trees with some spruce admixture; moist and swamp forests composed of alder in carr forests and ash with alder in riparian forests; and coniferous forests dominated by pine and spruce (Faliński, 1986). These forest communities are formed by various tree species occurring in a mosaic pattern, influenced by local microhabitat variability. The region exhibits an exceptionally rich animal diversity, with birds being the most numerous groups of breeding vertebrates, with over 110 breeding species documented (Tomiałojć & Wesołowski, 2004). The bird communities in oak‐lime‐hornbeam and ash‐alder habitats are particularly species‐rich compared to other forest types (Wesołowski et al., 2022). For our study, we conducted research in deciduous stands within BNP, utilizing six sample plots (three plots in oak‐lime‐hornbeam stands and three in ash‐alder stands).

### 2.2 Data collection

We conducted the survey from May to June 2020 and 2021 (all controls were made in full leaves development). Within each sample plot, we randomly selected 16 points, separated by at least 200 m (96 points in total). On each observation point, we made four visits: two mornings (starting after sunrise and continuing for up to 3 h, one in the early season and the second one in the late season) and two evenings (starting up to 3 h before sunset, one in the early season and the second one in the late season). Such differentiation of observations aimed to capture birds with varying day activity and different migration times. That way we aimed to capture a whole variability of species in study sites. An observer spent 15 min at a point, using 10 × 50 binoculars to determine birds' position on trees, by direct observation. We recorded the birds' position in the first spotted location on a tree. Each individual was identified at the species level.

**FIGURE 2:**
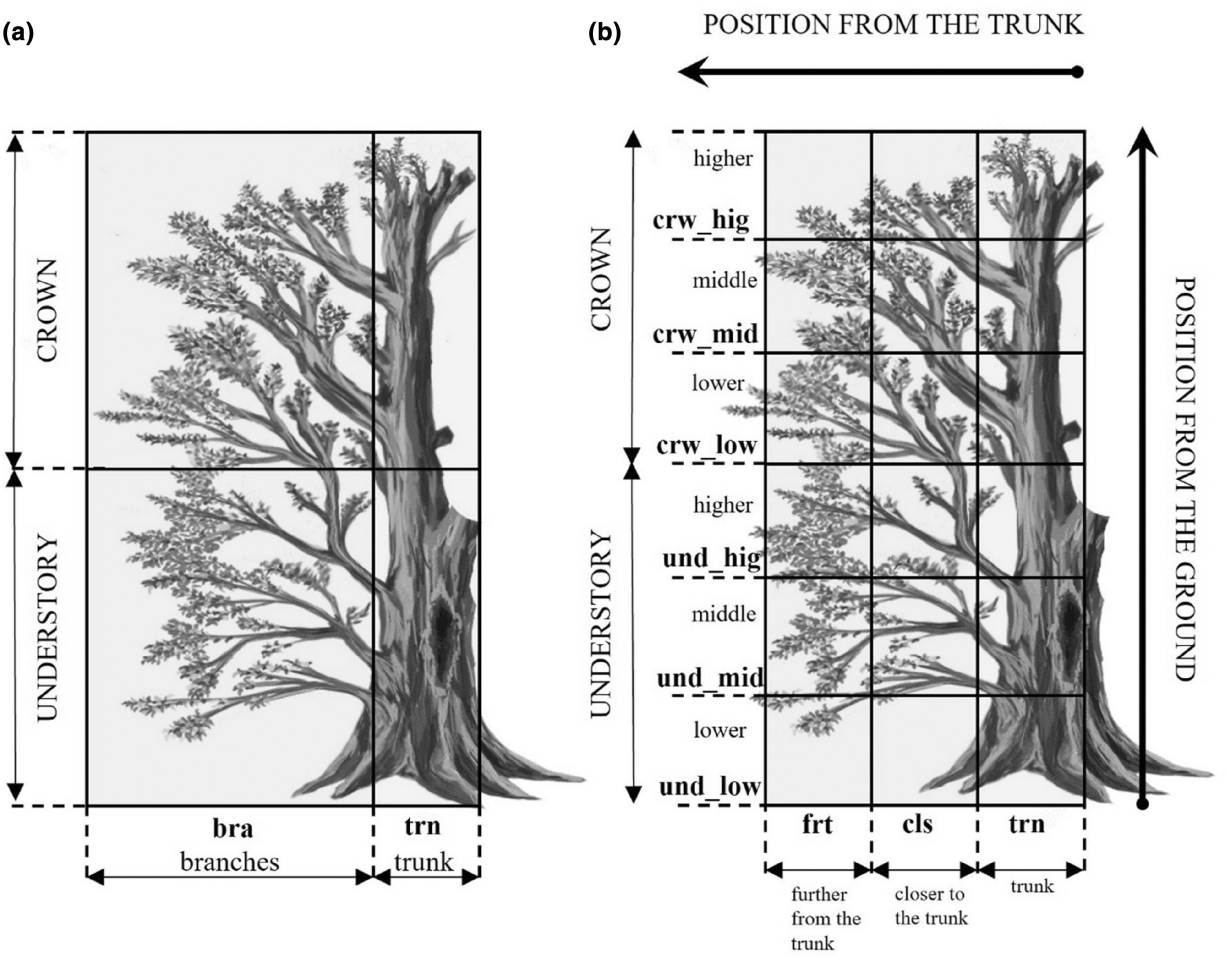
Sector division on the tree by the position from the ground and the position from the trunk. (a) Contains four main sectors, like understory, crown, trunk, and branches, (b) is more detailed, containing 18 sectors, the position from the trunk (further from the trunk, closer to the trunk, trunk), the position from the ground (lower understory, middle understory, higher understory, lower crown, middle crown, and higher crown).

It is hard to evaluate the real height of a tree in the field. To avoid inaccurate data we create a form, that could be universal for every tree shape, without paying attention to tree height. Each tree could be divided into parts with a main crown and a main trunk. We define a tree crown as the place where a tree starts to divide into smaller boughs. The main crown could be easily divided horizontally (a position from the ground) into three main levels: lower, middle, and higher. Such a method approach is easier in the field, we do not have to know any metrical data. Moreover, having a reference to a main trunk amongst the tree crown, we could make a quick division into three parts vertically (position from the trunk): main trunk amongst the tree crown, branches that are closer to the trunk, and branches that are further from the trunk (if we are looking at the branch on the tree, we dive it into two parts, that closer and that further from the trunk). Analogously, the understory level branches could be divided horizontally and vertically (Figure 2b). In Białowieża Forest there are a lot of trees with side branches growing from the main trunk below to the main crown level. However, not every tree could always have the same parts. Some of them do not have all side branches, or a tree crown could be partially damaged. Moreover, not every part of the tree is utilized by birds. Thus, we decided to make analyses in the simplified form of tree division (Figure 2a), to obtain the simpler pattern of tree utilization by birds.

We are aware of probable observation bias, that could result from random effects as observer experience or visibility level in different habitats. We should consider that in some parts of the forest, due to dense undergrowth, it was easier to see the tree canopies than what was happening at the base of the trees. Conversely, in areas with sparse undergrowth, birds were often visible on the tree trunks and forest floor. There were also sections of the forest with gaps where the canopy coverage was sparse, and the tree crowns were clearly visible. Hence, there were numerous observation points to conduct observations at different levels of visibility.

We analyzed spatial niche segregation of birds for all tree species combined, and here we focused on sectors, and our research unit was a sector on all trees within tetrads of points within the plot. Due to the absence of birds at many observation points we decided to aggregate the observations. We merged observations from the four nearest points within a particular plot into tetrads, providing a sufficient number of non‐zero observations and simultaneously allowing for including within‐plot variability. That way within each plot there are four tetrads, aggregating four points each (in total 24 tetrads) (Figure S1).

### 2.3 Statistical analyses

All bird species observation was used for statistical analyses to examine differences in species richness. Moreover, for 10 of the most frequently observed species, analyses of probability of occurrence across tree sectors were made. We chose these species because a sufficient number of observations ensured meaningful estimates of trends. As many species were represented by few observations, statistical models predicted zero probabilities of their occurrence due to an overwhelming number of negative observations, despite different methods of zero‐inflation accounting during preliminary analyses. To explore similarities in species composition of birds (*n* = 49) amongst sites representing tree sectors position from the ground (associated with tree crowns and understory) and trunk (sectors associated with trunks and branches) we employed ordination techniques. Based on a gradient length (4.93 SD) obtained from the preliminary detrended correspondence analysis (DCA *vegan::decorana()* function) (Oksanen et al., 2022) of species richness data, we decided to use DCA ordination as the most suitable approach for illustrating differences in the composition of bird species assemblages amongst the tetrads of points representing particular positions of tree sectors.

To examine how the position of tree sectors (situated in crowns and understory, as well as associated with trunks and branches) influences species richness of birds in tetrads of points, we performed the generalized linear mixed model (GLMM; *glmmTMB::glmmTMB()* function) (Brooks et al., 2022). In this GLMM we defined the position of tree sectors as a fixed effect, and the tetrad of points nested in the plot as a random factor, accounting for potential effects linked with the spatial distribution of sites in the study area, and variability in tree stand species composition. We built similar GLMM to assess differences in species richness of birds amongst tetrads of points representing 18 tree sectors. A nonparametric dispersion test implemented in the *DHARMa::testDispersion()* function (Hartig, 2022) revealed relatively low values of dispersion (~1.00) in case of both GLMMs, allowing us to assume no overdispersion of GLMMs, and Poisson distribution of response variable. Using the *MuMIn::r.squaredGLMM()* function (Bartoń, 2023), for both GLMMs we calculated the marginal ($R_{m}^{2}$) and conditional ($R_{c}^{2}$) coefficients of determination. While $R_{m}^{2}$ reveals how much variance is explained only by random effects, $R_{c}^{2}$ illustrates the amount of variance explained by both random and fixed factors. Thus, the disparity between $R_{c}^{2}$ and $R_{m}^{2}$ shows the amount of variance explained only by a fixed factor.

To reveal the relationships between the presence/absence of individual bird species and tetrads of points representing tree sectors position from the ground (associated with tree crowns and understory) and trunk (sectors associated with trunks and branches), we used GLMMs with a binomial distribution of response variables (particular species presence), and a tetrad of points nested in the plot as a random factor. In these GLMMs position from the ground and position from the trunk were fixed effects, and site identifier (i.e., identifier of tetrad nested in plot identifier) was a random intercept. We performed the similar GLMMs to assess the probability of occurrence of particular bird species regarding tetrads of points representing each of the 18 tree sectors. In these GLMMs tree sector was a fixed effect, and site identifier (i.e., identifier of tetrad nested in plot identifier) was a random intercept. In both procedures, we analyzed only those bird species that occurred at more than 40 points, reducing the number of tested birds from 49 to 10 (*Dendrocopos major*, *Parus major*, *Phylloscopus collybita*, *Sylvia atricapilla*, *Sitta europaea*, *Erithacus rubecula*, *Ficedula albicollis*, *Turdus merula*, *Fringilla coelebs*, and *Coccothraustes coccothraustes*).

Following the American Statistical Association guidelines we did not account for *p‐*values as a tool for assessment of the significance of results, as a strong sample‐size dependency of *p‐*values may generate a risk of treating biologically interpretable patterns as statistically not significant (Nakagawa & Cuthill, 2007; Wasserstein & Lazar, 2016). Instead, via the usage of Tukey's post hoc test with studentized adjustment for multiple hypothesis testing implemented in the *emmeans::emmeans()* function (Lenth, 2022), we focused more on the effect sizes than *p‐*values regarding all GLMMs. All statistical analyses we performed using R version 4.2.0 “Vigorous Calisthenics” (R Core Team, 2022).

## 3 RESULTS

**FIGURE 3:**
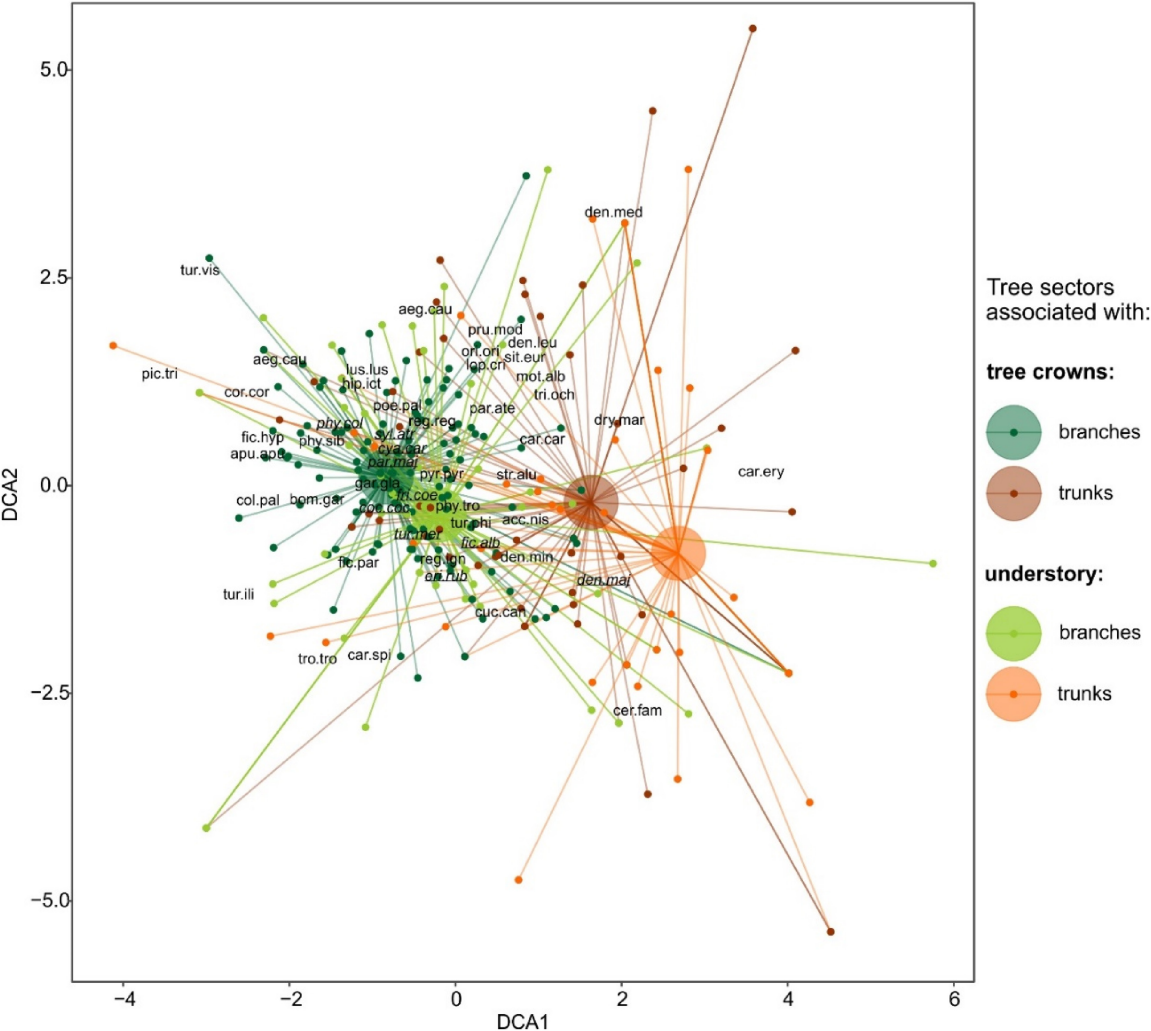
Results of detrended correspondence analysis (DCA) ordination illustrating differences in species composition of all bird species recorded in the study area (*n* = 49) amongst tree sectors position from ground (associated with tree crowns and understory) and trunk (sectors associated with trunks and branches). Points represent sites. Large circles are centroids of sites representing a particular position of tree sectors (connected with points using lines). Coordinates of the most common (recorded in more than 40 occurrences) bird species (*n* = 10) in the study area are presented as *italicized* and underlined acronyms. For full species names see Table S1.

During the 2 years of the study, 1622 observations of individuals belonging to 49 bird species were collected. The DCA ordination, depicting tetrads of points representing four tree sectors, and bird assemblages structure, showed compositional dissimilarity between tetrads representing different sectors reflected in their position in regards to trunks, branches, crowns, and understory, expressed in three disparate groups (Figure 3) (large, dark green dot, representing tree crown branches, with close large, light green dot representing understory branches constitute one group; large, dark orange dot, representing tree crown trunk constitutes the second group; large, light orange dot representing understory trunk constitutes the third group). We observed the most numerous group of birds within tetrads representing tree crowns and understory branches. Tetrads representing branches agglomerated most of the BNP bird community. We observed that the tree crown trunk and the understory trunk are the least numerous in bird species (Figure 3).

**FIGURE 4:**
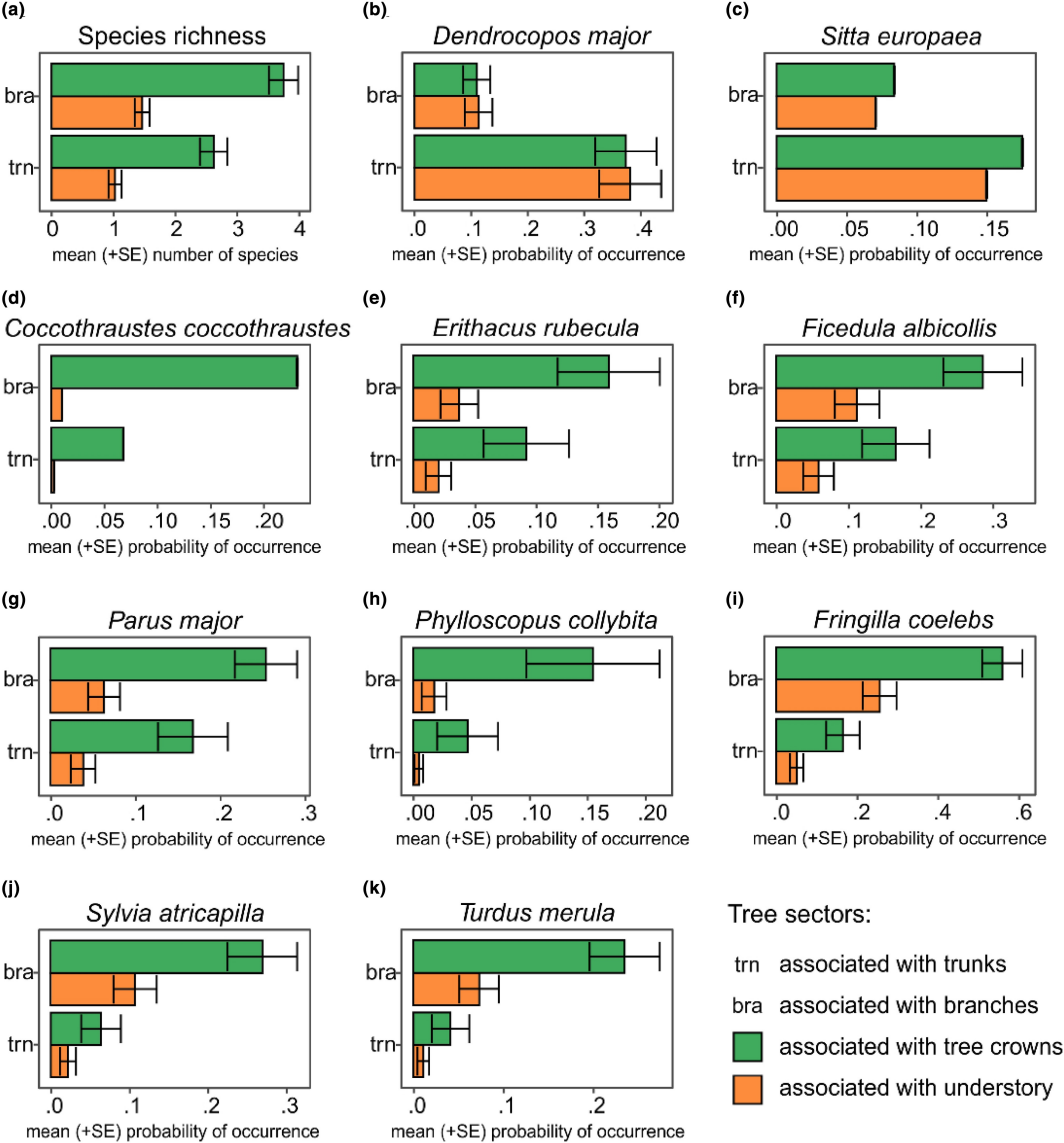
Mean (+SE) species richness and probabilities of occurrence of the most common (recorded in more than 40 occurrences) bird species (*n* = 10) in the study area per position of tree sectors from ground (sectors associated with tree crowns and understory) and trunk (sectors associated with trunks and branches) predicted using generalized linear mixed models assuming Poisson distribution (in case of species richness) and binomial distribution (in case of particular bird species occurrence probability). For model parameters see Table 1.

The highest species richness we observed on tetrads representing branches in the crown level of a tree (Figure 4a, Table 1). For *D. major* and *S. europaea*, the highest probability of occurrence (hereafter POC) was on trunks (both in the crown and understory sectors) (Figure 4b,c, Table 1). We found an opposite pattern for *C. coccothraustes*, *E. rubecula*, *F. albicollis*, *P. major*, and *P. collybita*, *F. coelebs*, *S. atricapilla*, and *T. merula*. The highest probability of occurrence, for these species, was on branches associated with the crown (Figure 4d–k, Table 1).

**TABLE 1:** Parameters of generalized linear mixed models testing for effects of position of tree sectors from ground (Pos. ground) and trunk (Pos. trunk) on species richness of birds (Poisson distribution) and the occurrence of the most common (recorded in more than 40 occurrences) bird species (*n* = 10) in the study area (binomial distribution).

Response variable	Mixed model parameters					ANOVA			
	RE variance	RE SD	$R_{m}^{2}$	$R_{c}^{2}$	$R_{c}^{2}-R_{m}^{2}$	Predictor	*χ* ^2^	df	*p*
Species richness	0.047	0.218	.343	.41	.067	Pos. ground	177.823	1	<.001
						Pos. trunk	29.399	1	<.001
*Dendrocopos major*	0.164	0.405	.137	.178	.041	Pos. ground	0.016	1	.898
						Pos. trunk	37.529	1	<.001
*Coccothraustes coccothraustes*	0.449	0.67	.472	.535	.063	Pos. ground	1,445,910	1	<.001
						Pos. trunk	253,383	1	<.001
*Erithacus rubecula*	0.886	0.941	.146	.327	.181	Pos. ground	16.56	1	<.001
						Pos. trunk	2.647	1	.103
*Ficedula albicollis*	0.827	0.909	.097	.279	.182	Pos. ground	18.091	1	<.001
						Pos. trunk	5.692	1	.017
*Fringilla coelebs*	0.305	0.552	.250	.314	.064	Pos. ground	29.251	1	<.001
						Pos. trunk	37.658	1	<.001
*Parus major*	0.034	0.184	.178	.186	.008	Pos. ground	22.636	1	<.001
						Pos. trunk	2.574	1	.108
*Phylloscopus collybita*	1.953	1.397	.245	.526	.281	Pos. ground	23.375	1	<.001
						Pos. trunk	8.108	1	.004
*Sitta europaea*	0.404	0.636	.043	.147	.104	Pos. ground	6387.9	1	<.001
						Pos. trunk	129,083	1	<.001
*Sylvia atricapilla*	0.318	0.564	.208	.278	.070	Pos. ground	13.637	1	<.001
						Pos. trunk	16.120	1	<.001
*Turdus merula*	0.144	0.380	.278	.308	.380	Pos. ground	14.633	1	<.001
						Pos. trunk	13.417	1	<.001

*Note*: Differences in mean (+SE) species richness and mean (+SE) probabilities of occurrence of bird species amongst the position of tree sectors from ground and trunk are presented in Figure 4.

Abbreviation: RE, random effect.

**FIGURE 5:**
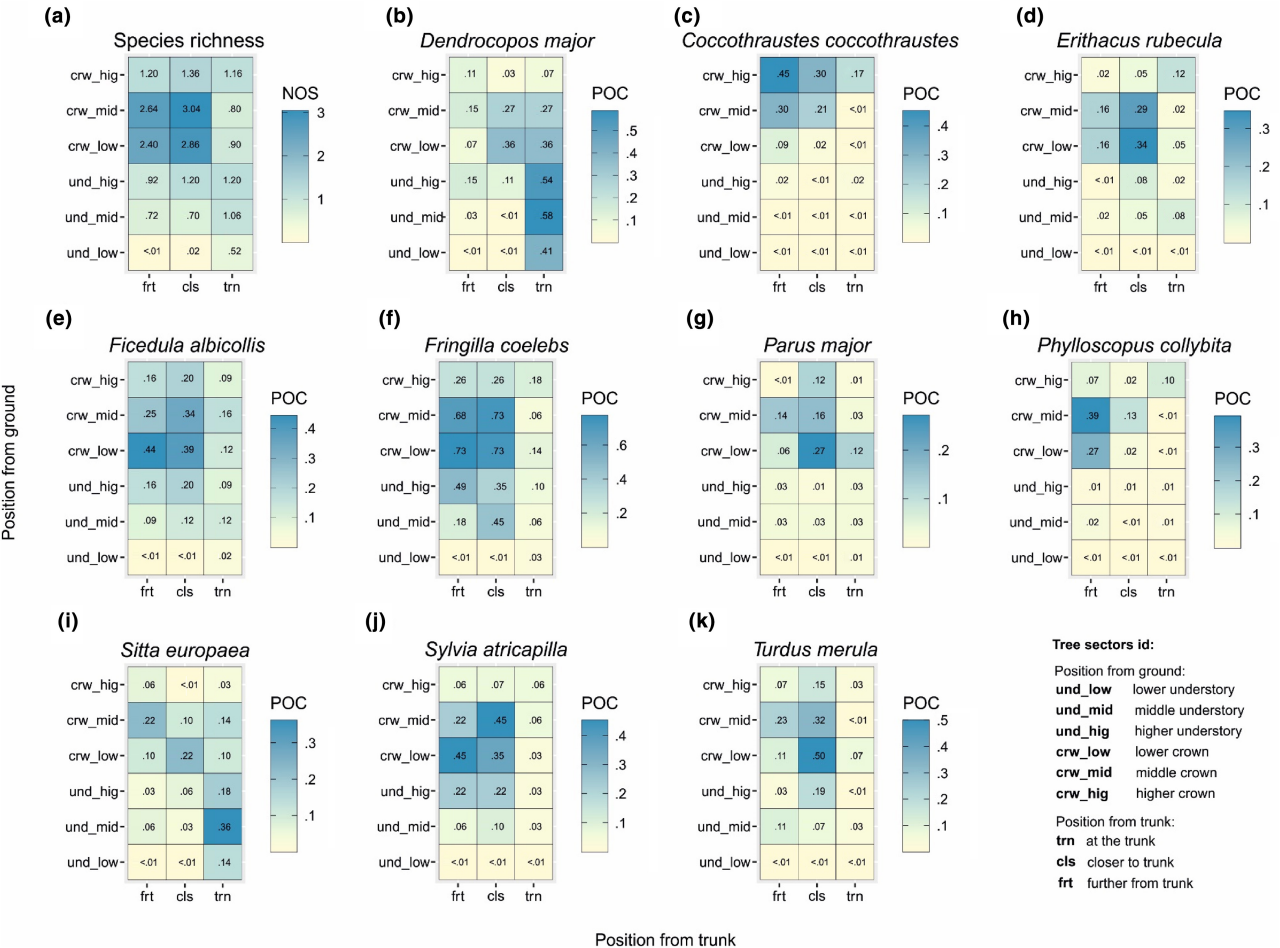
Heat maps showing differences in mean (+SE) species richness and probabilities of occurrence of the 10 most common bird species in the study (*Dendrocopos major*, *Coccothraustes coccothraustes*, *Erithacus rubecula* and *Ficedula albicollis*, *Fringilla coelebs*, *Parus major*, *Phylloscopus collybita*, *Sitta europaea*, *Sylvia atricapilla*, and *Turdus merula*) area amongst 18 tree sectors predicted using generalized linear mixed models assuming Poisson distribution (in case of species richness) and binomial distribution (in case of particular bird species occurrence probability). Coordinate (*X*) represent the position of sectors from trunk. Coordinate (*Y*) represent the position of sectors from ground level. NOS, number of species; POC, probability of occurrence. For model parameters see Table 2.

Heatmaps showing the probability of occurrence on each of 18 tree sectors (and the number of species in case of species richness plot), inform about tree area utilization by particular bird species (Figure 5). Regarding all 49 species combined, the highest species richness we observed on sectors located in the middle crown closer to the trunk and on the lower crown closer to the trunk (Figure 5a, Table 2). Similar broad pattern of tree utilization we found amongst *F. albicollis*, *F. coelebs*, and *S. atricapilla* (Figure 5e,f,j, Table 2). The narrowest tree utilization performed *C. coccothraustes* and *P. collybita* (Figure 5c,h, Table 2). Comparable pattern focused on side branches exhibited *E. rubecula*, *P. major*, and *T. merula* (Figure 5d,g,k, Table 2). Tree utilization pattern focused on tree trunk we found amongst *D. major* and *S. europaea* (Figure 5b,i, Table 2).

**TABLE 2:** Parameters of generalized linear mixed models (GLMMs) showing differences in species richness of birds (Poisson distribution) and the occurrence of the most common (recorded in more than 40 occurrences) bird species (*n* = 10; binomial distribution) in the study area amongst 18 tree sectors.

Response variable	Mixed model parameters					ANOVA		
	RE variance	RE SD	$R_{m}^{2}$	$R_{c}^{2}$	$R_{c}^{2}-R_{m}^{2}$	*χ* ^2^	df	*p*
Species richness	0.047	0.218	.974	.976	.002	279.59	17	<.001
*Dendrocopos major*	0.295	0.544	.886	.892	.006	46.538	17	<.001
*Coccothraustes coccothraustes*	0.781	0.883	.888	.896	.008	22.635	17	.161
*Erithacus rubecula*	1.232	1.110	.841	.858	.017	28.079	17	.044
*Ficedula albicollis*	1.004	1.002	.748	.783	.035	27.067	17	.057
*Fringilla coelebs*	0.567	0.753	.868	.882	.014	83.120	17	<.001
*Parus major*	0.118	0.344	.846	.848	.002	41.617	17	<.001
*Phylloscopus collybita*	3.509	1.873	.869	.901	.032	36.671	17	.003
*Sitta europaea*	0.551	0.742	.805	.816	.011	21.858	17	.190
*Sylvia atricapilla*	0.516	0.718	.835	.845	.010	3,342,217.0	17	<.001
*Turdus merula*	0.255	0.505	.872	.875	.003	31.811	17	.015

*Note*: Differences in mean (+SE) species richness and mean (+SE) probabilities of occurrence of bird species amongst 18 tree sectors are presented in Figure 5.

Abbreviation: RE, random effect.

## 4 DISCUSSION

### 4.1 Species richness distribution

We observed the highest species richness of birds at the crown level, as they predominantly occupied branches, while the various zones of the trunk were mainly inhabited by a few specialized species (e.g., *D. major* or *S. europaea*). Conversely, the lower sections of both the crown and trunk exhibited the lowest species richness. This distribution pattern could be attributed to various factors, with food availability being one of the most significant (Villard & Foppen, 2018). Our study documented a range of behaviors, with approximately 30% of them associated with foraging (O. Karpińska, unpublished data). During the breeding season, nearly 90% of birds in BNP deciduous stands rely on insects as their primary food source (Tomiałojć et al., 1984). Moreover, half of them gather invertebrates from leaves and twigs (Wesołowski, 2003). It appears that the highest concentration of bird diversity beneath tree branches coincides with the richest food supply, thus attracting predominantly insectivorous species, for example, the nestlings of several bird species like *T. merula*, *F. albicollis*, *Dendrocopos medius*, *Phylloscopus sibilatrix*, *Ficedula parva*, *Cyanistes caeruleus*, *P. major*, *Poecile palustris*, *S. europaea*, *E. rubecula* primarily feed on florivorous caterpillars (e.g., Tomiałojć, 1993; Walankiewicz, 2006; Wesołowski et al., 2019). Additionally, lots of bird species include in their diet spiders (e.g., Wesołowski & Neubauer, 2017), which could constitute more than 20% of the diet (e.g., Mitrus et al., 2010), and flying insects (e.g., Maziarz & Wesołowski, 2010).

The vertical and horizontal stratification of birds within trees is a widely recognized phenomenon, particularly in tropical forests (Walther, 2002). Arthropods, which constitute an essential component of the diet for many bird species (Wesołowski, 2003), also exhibit stratification patterns (Ulyshen, 2011). This stratification is likely influenced by factors such as the arrangement of leaves on branches and the presence of living and dead twigs of varying sizes (Parker & Brown, 2000). Tucker and Evans (1997) state that within the tree, the majority of birds engage in foraging activities within the canopy, while a smaller proportion can be found on the trunk (requiring appropriate adaptations such as sharp claws and beaks). Our study confirmed that *D. major* demonstrates a strong association with tree trunks. This adaptation is supported by its morphology (Gorman & Kokay, 2004). In contrast, small foliage‐gleaning species such as *C. caeruleus* or *P. major* exhibit different adaptations. They employ their toes to grip twigs and utilize a narrow, pointed bill for capturing insects (Cramp & Perrins, 1993). The vertical distribution of arthropods is influenced by various factors that depend on several issues including habitat characteristics, sun exposure, humidity, and temperature (Wagner et al., 2003).

In our study, a significant portion of observed mating behavior involved male vocalization. We observed numerous instances of males singing within dense foliage located in the mid‐crown or understory levels (unpublished data). Certain species, such as *E. rubecula*, often select dense spruce vegetation as their preferred singing location, making them challenging to spot (Cramp & Brook, 1988). The dense foliage found in crowns and understory areas offers birds a suitable hiding place against predators during activities such as foraging, mating, roosting, or grooming. Singing beneath dense foliage provides birds with greater safety compared to singing in open areas. Moreover, the dense habitat does not impede the propagation of their vocalizations. Forest birds have adaptations that enable them to communicate effectively within complex environments. They typically sing at lower maximal frequencies compared to birds in open habitats (Wiley, 1991).

Not only foraging and mating behavior could influence the bird distribution on trees. There are several factors determining tree niche partitioning like predation and mechanisms of its avoidance, morphophysiological adaptation, or intraspecific competition (Villard & Foppen, 2018). However, in our studies, which consider multiple species together as well as tree species collectively, it is not possible to focus on other factors influencing the distribution of birds on a tree. Here we present different distribution patterns represented by the 10 most numerous bird species in BNP.

### 4.2 Tree usage patterns

We could distinguish several patterns of tree utilization by birds in BNP: broad tree use pattern with crown specialization (*F. albicollis*, *S. atricapilla*, and *F. coelebs*), broad tree use pattern with trunk specialization (*D. major*, *S. europaea*), middle tree use pattern with crown specialization (*E. rubecula*, *P. major*, *T. merula*), and narrow tree use pattern with crown specialization (*P. collybita*, *C. coccothraustes*). Despite some overlapping of niches in certain sectors and the influence of specific filters on certain species (such as bark foragers), the differentiation of niches in three dimensions appears to be the primary mechanism shaping the co‐occurrence of birds on trees within the primary forest. The branches located in the middle and lower parts of the crown serve as hotspots for bird diversity, with a concentration of insectivorous species. This is likely due to the higher abundance of various food sources within the crown. The patterns of tree usage by individual bird species differ, indicating the avoidance of interspecific competition for food, nest sites, and roosts. Previous studies have already suggested weak competitive interactions for nest sites and food amongst individual species in the Białowieża Forest due to a high abundance of these resources on the one hand and high predation pressure on the other (e.g., Walankiewicz, 1991; Wesołowski, 2003, 2007). However, our research is the first to analyze a significant portion of the forest bird community in relation to all available tree resources. The findings from our study conducted in a pristine condition can serve as a valuable model for studying interspecific interactions in forests that have been transformed by human activities. Further research focusing on the distribution of all tree resources is necessary.

## 5 CONCLUSIONS

In summary, our study revealed distinct patterns of bird distribution within the BNP, particularly in relation to tree utilization and the vertical stratification of avian species. The observed concentration of bird diversity in the middle and lower crown branches, associated with the abundance of insectivorous species, underscores the influence of food availability on bird distribution. Furthermore, while our research highlighted limited interspecific competition for resources within this pristine forest, it signifies the need for further investigation into interspecific interactions, emphasizing the value of this study as a foundational model for understanding such dynamics in human‐altered forest environments.

## AUTHOR CONTRIBUTIONS

**Oliwia Karpińska:** Conceptualization (lead); data curation (equal); formal analysis (equal); investigation (lead); methodology (lead); project administration (lead); resources (equal); software (equal); visualization (supporting); writing – original draft (lead); writing – review and editing (lead). **Katarzyna Kamionka‐Kanclerska:** Conceptualization (equal); investigation (equal); methodology (equal); writing – original draft (supporting); writing – review and editing (equal). **Patryk Czortek:** Data curation (lead); formal analysis (lead); methodology (equal); resources (equal); software (lead); supervision (equal); validation (equal); visualization (lead); writing – original draft (equal); writing – review and editing (lead). **Marcin K. Dyderski:** Data curation (equal); formal analysis (equal); funding acquisition (equal); software (equal); validation (equal); writing – review and editing (equal). **Dorota Czeszczewik:** Conceptualization (equal); funding acquisition (equal); investigation (equal); methodology (equal); project administration (equal); supervision (lead); validation (equal); writing – review and editing (lead).

## FUNDING INFORMATION

This research was supported by the University of Natural Sciences and Humanities in Siedlce and the Institute of Dendrology, Polish Academy of Science. MKD acknowledges support from the Foundation for Polish Science (FNP) from the START scholarship.

## CONFLICT OF INTEREST STATEMENT

The authors declare that they have no known competing financial interests or personal relationships that could have appeared to influence the work reported in this paper.

## Supporting information


Figure S1

Table S1

## Data Availability

The dataset with novel code is available at https://doi.org/10.48370/OFD/D7PPAY (Karpińska, 2023).
