# Supplementary material for: Patterns of avian tree usage in the primeval temperate forests of Białowieża National Park
Source: Ecol Evol. 2024 Apr 15;14(4):e11138. doi: 10.1002/ece3.11138 (PMC11019296; doi:10.1002/ece3.11138)
Supplement: Supplementary file 1 — Figure S1 [file ECE3-14-e11138-s002.docx]

**Patterns of avian tree usage in the primeval temperate forests of Białowieża National Park**

Supplementary material

Schematic sample plot Schematic observation points

Tetrad of the four nearest points within a particular plot

Fig. S1. Scheme of observation point merging methodology. Due to the absence of birds at many observation points, we decided to aggregate the observations. We merged observations from the four nearest points within a particular plot into tetrads, providing a sufficient number of non-zero observations and simultaneously allowing for including within-plot variability. That way within each plot there are four tetrads, aggregating four points each (in total 24 tetrads within six plots).

While a single data point (site) including all spatiotemporal variables would be defined as a combination of: Year × sample plot × point × day time × season time × tree × number of sectors

Including a particular number of levels for each variable:

(2 years) × (6 sample plots) × (16 points) × (2 day times) × (2 season time) × (tree number variable depending on the sample plot) × (18 sectors)

When the points are connected into tetrads and aggregated over time, a single site is included in the following design:

Year × sample plot × tetrad × tree × number of sector

Including a particular number of levels for each variable: (2 years) × (6 sample plots) × (4 tetrads) × (number variable depending on the sample plot) × (18 sectors)
