# Supplementary material for: Patterns of avian tree usage in the primeval temperate forests of Białowieża National Park
Source: Ecol Evol. 2024 Apr 15;14(4):e11138. doi: 10.1002/ece3.11138 (PMC11019296; doi:10.1002/ece3.11138)
Supplement: Supplementary file 2 — Table S1 [file ECE3-14-e11138-s001.docx]

Appendix S1

Table S1. Birds’ scientific names and their abbreviations (www.worldbirdnames.org/new/, version 23.08.2023).

|  |  |
| --- | --- |
| Latin name | Abbreviation |
| *Accipiter nissus* | *Acc.nis* |
| *Aeghitalos caudatus* | *Aeg.cau* |
| *Apus apus* | *Apu.apu* |
| *Bombycilla garrulus* | *Bom. gar* |
| *Carduelis carduelis* | *Car.car* |
| *Carduelis spinus* | *Car.spi* |
| *Certhia familiaris* | *Cer.fam* |
| *Coccothrauses Coccothrauses* | *Coc.coc* |
| *Columba palumbus* | *Col.pal* |
| *Corvus corax* | *Cor.cor* |
| *Cuculus canorus* | *Cuc.cuc* |
| *Cyanistes caeruleus* | *Cya.cae* |
| *Dendrocopos leucotos* | *Den.leu* |
| *Dendrocopos major* | *Den.maj* |
| *Dendrocoptes medius* | *Den.med* |
| *Dryobates minor* | *Dry.mar* |
| *Dryocopus martius* | *Dry.min* |
| *Erithacus rubecula* | *Eri.rub* |
| *Ficedula albicollis* | *Fic.alb* |
| *Ficedula hypoleuca* | *Fic.hyp* |
| *Ficedula parva* | *Fic.par* |
| *Fringilla coelebs* | *Fri.coe* |
| *Garrulus glandarius* | *Gar.gla* |
| *Hippolais icternia* | *Hip.ict* |
| *Luscinia luscinia* | *Lus.lus* |
| *Motacilla alba* | *Mot.alb* |
| *Muscicapa striata* | *Mus.str* |
| *Oriolus oriolus* | *Ori.ori* |
| *Parus major* | *Par.maj* |
| *Periparus ater* | *Per.ate* |
| *Phylloscopus collybita* | *Phy.col* |
| *Phylloscopus sibilatrix* | *Phy.sib* |
| *Phylloscopus trochilus* | *Phy.tro* |
| *Picoides tridactylus* | *Pic.can* |
| *Picus canus* | *Pic.tri* |
| *Poecile palustris* | *Poe.pal* |
| *Prunella modularis* | *Pru.mod* |
| *Regulus ignicapilla* | *Reg.ign* |
| *Regulus regulus* | *Reg.reg* |
| *Sitta europaea* | *Sit.eur* |
| *Strix aluco* | *Str.alu* |
| *Sturnus vulgaris* | *Stu.vul* |
| *Sylvia atricapilla* | *Syl.atr* |
| *Tringa ochropus* | *Tri.och* |
| *Troglodytes troglodytes* | *Tro.tro* |
| *Turdus merula* | *Tur.mer* |
| *Turdus philomelos* | *Tur.phi* |
| *Turdus viscivorus* | *Tur.vis* |
